# Letter from G. Y. Shirley, Dentist, to St. Louis Editor

**Published:** 1848-07

**Authors:** G.Y. Shirley

**Affiliations:** Den. Sur.


### *Letter from J. Y. Shirley. Dentist, to St. Louis Editor.*

Jacksonville, May, 1848.

Dr. B. B. Brown,

*Dear Sir*:—There is perhaps nothing appertaining to the Dental profession involving more importance to the patient, and demanding more skill of the operator, than the construction of artificial palates and obturators, so as to remedy the defects for which they are intended. To see one of our race, wearing the human form divine, and yet, that form so marred as to render the possessor an object of disgust to himself, and of sympathy to his friends, excites in the professional man emotions like those possessed by Him who came to earth, a lost world to save. And, though the operator cannot perform a *miracle* in this matter, yet he can, in many cases, render a benefit to the deformed and afflicted, compared to which, silver and gold are as dross.

As you honored me with an invitation to contribute something for the Dental Register of the West, I have determined to send you a short history of one of those cases to which I have alluded. Not that much more important operations have never been published, but, because they are of rare occurrence, and hence, I feel it a duty to cast in something, if but a mite, in order to interest the the profession, and if possible advance the cause. Leaving you to be the judge of its value, I proceed to the case. Joseph, aged 17 years, son of Mr. B. C. Johnson of Palmyra, Mo., lost, at the age of three years, the anterior portion of the superior maxillary bone of the left side, and the corresponding half of the upper lip, in consequence of the abuse of mercurial medicine. The mouth and left nostril were thus constantly exposed to view. His physiognomy was greatly changed and rendered even hideous, while his articulation was necessarily very imperfect. On March 9th, 1845, Dr. D. Prince, Professor of Surgery in the Medical Department of Ill. College, undertook the case by dissecting the fragmentery lip of the right side freely from its bony attachments, and also releasing the cheek of the left side from its connection to the bone beneath,—-removing its opposite edges with a bistoury upon a wooden spatula, as for hair-lip, and bringing the incised edges together by interrupted surtures. Thus the nnion was perfect, drawing, of course the left angle of the mouth too far toward the median line. Thus far there was an improvement, but still a communication was left between the mouth and left nostril. On Oct., 29th, 1847, the Doctor operated after the manner of Mutter, to extend the oral opening on the left, by making two incisions an inch in length, parallel with each other, and one fourth of an inch apart, removing the skin and muscular substance, and then dividing the muscular membrane by a simple iucision, making a mucous covering for the (intended) incisores, and preventing, as far as possible a diminution of the size of the mouth by the granulatious at the angle. The mouth was thus made nearly symmetrical, but the communication between the mouth and nostril still remained. On Nov. 15, 1847, two incisions were made opposite the opening from the mouth to the nostril, removing a triangular portion without completely detaching it, scarifying the surface bounding the orifice, thrusting a ligature through the triangular slip—carrying it upwards and forwards through the nostril by which the orifice was filled. Thc lip was then closed by interrupted sutures as before with perfect union. The lip however appeared too flat upon the affected side, from the want of an alveolus and teeth to remedy which he referred the patient to me. I extracted several teeth, filled such as could be saved, gave him appropriate remedies for his gums, and advised him to defer any further operation for several weeks. After a few days his anxiety to return home became so great, that he insisted I should proceed, and, if his teeth did not do well, he would return, to which I consented. I proceeded to obtain the cast of the superior arch in the usual way, except the wax impression was made to fill the arch entire, and finding the parts which had been brought in to fill the cleft palate necessarily very irregular, and not yet completely united—constructed a complicated artificial palate, by which I mean, a plate to cover the soft parts alluded to, and attach the artificial teeth by extending it posteriorly in an oblong form, and anteriorly so far as to supply the lost portion of the alveolus, that when the operation should be introduced, the lip might be elevated to its wanted position. This was made of 20 carat gold, and one third thicker than usual for artificial purposes, to give more permanent support to the teeth, and that it might not yield to pressure made upon the palatal portion when there was no bone. The central incisores, left cuspidate and first bicusped were wanting. I proceeded to supply them, leaving out the bicusped, as the parts were somewhat contracted. Two clasps were then made of 18 carat gold, taking hold of the first molar on the right, and remaining bicusped on the left. The crowns of these teeth were so low they could not be of much use, in addition to which the plate was adjusted upon the suction principle. This artificial contrivance was introduced in presence of Judge D. P. Henderson, at whose house Mr. Johnson stayed. He called upon him to pronounce several words involving the dental sounds, which he did very well. He said he had several times tried him with the same words which he could not pronounce so as to be understood by a stranger. The young man turned to the glass and expressed himself much pleased with his persoual improvement. I have heard from him several times recently through his friends, to whom he expresses himself much pleased with the important services rendered him by Professor Prinee and myself.

Very Respectfully, G. Y. SHIRLEY, Den. Sur.

